# Investigating Nutrient Limitation Role on Improvement of Growth and Poly(3-Hydroxybutyrate) Accumulation by *Burkholderia sacchari* LMG 19450 From Xylose as the Sole Carbon Source

**DOI:** 10.3389/fbioe.2019.00416

**Published:** 2020-01-08

**Authors:** Edmar R. Oliveira-Filho, Jefferson G. P. Silva, Matheus Arjona de Macedo, Marilda K. Taciro, José Gregório C. Gomez, Luiziana F. Silva

**Affiliations:** Department of Microbiology, Institute of Biomedical Sciences, University of São Paulo, São Paulo, Brazil

**Keywords:** xylose, *Burkholderia sacchari*, poly(3-hydroxybutyrate), bioreactors, biopolymers

## Abstract

*Burkholderia sacchari* LMG19450, a non-model organism and a promising microbial platform, was studied to determine nutrient limitation impact on poly(3-hydroxybutyrate) [P(3HB)] production and bacterial growth from xylose, a major hemicellulosic residue. Nitrogen and phosphorus limitations have been studied in a number of cases to enhance PHA accumulation, but not combining xylose and *B. sacchari*. Within this strategy, it was sought to understand how to control PHA production and even modulate monomer composition. Nitrogen-limited and phosphorus-limited fed-batch experiments in bioreactors were performed to evaluate each one's influence on cell growth and poly(3-hydroxybutyrate) production. The mineral medium composition was defined based on yields calculated from typical results so that nitrogen was available during phosphorus limitation and residual phosphorus was available when limiting nitrogen. Sets of experiments were performed so as to promote cell growth in the first stage (supplied with initial xylose 15 g/L), followed by an accumulation phase, where N or P was the limiting nutrient when xylose was fed in pulses to avoid concentrations lower than 5 g/L. N-limited fed-batch specific cell growth (around 0.19 1/h) and substrate consumption (around 0.24 1/h) rates were higher when compared to phosphorus-limited ones. Xylose to PHA yield was similar in both conditions [0.37 g_P(3HB)_/g_xyl_]. We also described *pst* gene cluster in *B. sacchari*, responsible for high-affinity phosphate uptake. Obtained phosphorus to biomass yields might evidence polyphosphate accumulation. Results were compared with studies with *B. sacchari* and other PHA-producing microorganisms. Since it is the first report of the mentioned kinetic parameters for LMG 19450 growing on xylose solely, our results open exciting perspectives to develop an efficient bioprocess strategy with increased P(3HB) production from xylose or xylose-rich substrates.

## Introduction

Polyhydroxyalkanoates (PHA) are biopolyesters naturally produced and accumulated as intracellular granules by many Eubacteria and Archaea strains under unbalanced growth conditions (Lee, 1996; Koller et al., 2017). Considered as biocompatible and fully biodegradable (Keshavarz and Roy, 2010), these biopolymers are proposed as a valuable biomaterial presenting promising biomedical applications (Zhang et al., 2018). Although it has interesting characteristics, PHA production is still too expensive due to the prices of carbon sources, which represents up to 50% of the final production costs (Choi and Lee, 1997). This is one of the main obstacles in effectively introducing these biopolymers in the market.

The use of agroindustrial by-products as carbon feedstocks has been a strategy to produce PHA and other bioproducts in integrated biorefineries (Perlack, 2005; Silva et al., 2014; Aslan et al., 2016; Wang et al., 2018). Xylose, the second most abundant sugar in nature (Lachke, 2002), emerges as an exciting agricultural by-product to be explored as an inexpensive carbon source to produce PHA (Silva et al., 2014; Jiang et al., 2016). In Brazil, 633 × 10^6^ tons of sugarcane were processed in the 2017/2018 harvest (https://www.conab.gov.br/info-agro/safras/cana/boletim-da-safra-de-cana-de-acucar), representing 177 × 10^6^ tons of bagasse (Pessoa et al., 1997), part of which is burned to generate energy in the sugar and ethanol mills (Nonato et al., 2001). If submitted to an efficient hydrolysis treatment, as described by Paiva et al. (2009), this amount of bagasse would represent up to 430 × 10^5^ tons of xylose available to be converted into value-added bioproducts.

Since the present federal legislation in Brazil aims to gradually eliminate the burning step performed previously to harvest, more sugarcane residues are expected to be available to other industrial applications (Silva et al., 2014). In addition, the production of second-generation ethanol is still not feasible, considering that hemicellulosic sugars are still poorly fermented by ethanologenic yeasts and bagasse pretreatment high costs (dos Santos et al., 2016).

Among a diversity of PHA^+^ bacterial strains, *Burkholderia sacchari* LFM101 LMG19450^T^ (Brämer et al., 2001; Alexandrino et al., 2015) stands out for its ability to produce poly(3-hydroxybutyrate) [P(3HB)] from sucrose (Gomez et al., 1996, 1997). *B. sacchari* also metabolizes other carbohydrates: xylose, glucose, and arabinose (Brämer et al., 2001; Silva et al., 2004). Conveniently, *B. sacchari* is capable of incorporating other short- and medium-chain monomers as 3-hydroxyvalerate (3HV), 4-hydroxybutyrate (4HB), and 3-hydroxyhexanoate (3HHx) in the nascent polymeric chain, when supplied with co-substrates (Mendonça et al., 2014; Miranda De Sousa Dias et al., 2017).

One of the requirements for expressive PHA accumulation is carbon source excess combined with limitation of nutrients, such as nitrogen, phosphorus, iron, sulfur, magnesium, potassium, or oxygen (Schlegel et al., 1961). Therefore, the study of different nutritional limitations is an important factor to increase PHA concentration and content, as each nutrient limiting condition might have different effects on cell metabolism, growth, and PHA production. According to each strain, the best performance on polymer accumulation can be associated to one specific nutrient and must be determined to improve process of production.

Albeit important, there are only a few studies with phosphorus limitation as condition for PHA production available (Tu et al., 2019), most of which focused on PHA production from activated sludge (Chinwetkitvanich et al., 2004; Rodgers and Wu, 2010; Wen et al., 2010; Cavaillé et al., 2013; Tu et al., 2019). *Pseudomonas putida*, the model organism for medium-chain-length PHA production, was evaluated under different phosphorus limitation conditions, at various cultivations times, achieving increased PHA productivity, content, and concentration (Lee et al., 2000). Also, the initial phosphate concentration in *Ralstonia eutropha* fed-batch was shown as critical to achieve higher P(3HB) productivity and concentration values (Ryu et al., 1997). *Haloferax mediterranei* PHA production capacity was also evaluated under phosphorus limitation revealing promising results (Lillo and Rodriguez-Valera, 1990; Melanie et al., 2018).

An important factor to be considered when studying PHA production under phosphorus limitation is the fact that a previous study suggested that *B. sacchari* probably accumulates inorganic polyphosphate (Gomez et al., 1997). Polyphosphate accumulation in bacteria has been described to happen under several stress conditions, such as low pH, osmotic stress, or nutrient limitations, and might have various physiological functions: energy source, reservoir for phosphorus, among others (Kornberg et al., 1999). Additionally, some studies show that PHA and polyphosphate metabolism (production and utilization) are particularly linked, as evidenced by PHA depolymerase or polyphosphate kinase mutant strains of *R. eutropha* H16 (Tumlirsch et al., 2015) and *P. putida* KT2440 (Casey et al., 2013), which have differential PHA and polyphosphate production profiles.

In this work, the effects of nitrogen or phosphorus limitations in *B. sacchari* LFM101 growth and PHA biosynthesis using xylose as the sole source of carbon were evaluated. Also, the genes related to phosphorus consumption and polyphosphate were annotated in *B. sacchari* genome sequence. Results presented herein will be useful for the improvement of PHA homo- and copolymers production from xylose or xylose-rich agroindustrial by-products using *B. sacchari*, a promising microbial cell factory for bioproduction of value-added bioproducts, in the context of an integrated biorefinery (Nonato et al., 2001).

## Materials and Methods

### Microorganisms and Culture Media

*B. sacchari* LFM101 LMG19450^T^, recently reclassified as *Paraburkholderia sacchari* (Sawana et al., 2014), was used in this study as a platform for the production of PHA from xylose.

Lysogeny Broth (LB) (NaCl, 5 g/L; tryptone, 10 g/L; and yeast extract, 5 g/L) was used to grow *B. sacchari*. Seed cultures for fed-batch experiments were grown on mineral salts medium (MM1) with xylose as the sole carbon source (15 g/L). MM1 composition is detailed in Table 1. Flasks were incubated in a rotary shaker for 20 h at 30°C and 150 rpm.

**Table 1:** Composition of the media used in this work.

**Component**	**MM1**	**MM2 (N limited)**	**MM3 (P limited)**
**Medium composition (g/L)**			
Na_2_HPO_4_	3.50	–	–
KH_2_PO_4_	1.50	0.87	0.21
(NH_4_)_2_SO_4_	3.00	2.91	11.63
MgSO·7H_2_O	0.20	0.31	0.31
CaCl_2_·2H_2_O	0.01	0.01	0.01
Ammonium citrate iron	0.06	0.06	0.06
NaCl	–	1.00	1.00
Trace elements solution	1 ml/L	2 ml/L	2 ml/L

*Changes between MM2 and MM3 are in gray*.

*Trace elements solution composition was: H_3_BO_3_ (0.30 g/L); CoCl_2_·6H_2_O (0.20 g/L); ZnSO_4_·7H_2_O (0.10 g/L); MnCl_2_·4H_2_O (0.03 g/L); NaMoO_4_·2H_2_O (0.03 g/L); NiCl_2_·6H_2_O (0.02 g/L); CuSO_4_·5H_2_O (0.01 g/L)*.

Fed-batch/bioreactor experiments were carried out in two different mineral salts media: MM2 and MM3. MM2 composition was adapted to limit nitrogen availability and MM3 was adapted to limit phosphorus availability. Both media are detailed in Table 1. Xylose was sterilized separately and aseptically added to the culture media; the final concentration used was 15–20 g/L, which was sufficient for cell growth until the start of P(3HB) accumulation phase considering previously obtained yield data (Guamán et al., 2018).

### Culture Conditions

#### Fed-Batch Cultivations

For bioreactor assays, *B. sacchari* LFM101 was pre-incubated for 24 h at 30°C and 150 rpm in 1-L Erlenmeyer flasks with 200 ml of MM1 containing xylose (15 g/L). Two sets of fed-batch experiments were performed in two identical bioreactors (Applikon Biotechnology Inc., Delft, Netherlands) using a working volume of 3 L, at 30°C for up to 70 h. The first set was B101-X1 (nitrogen limitation) and B101-X2 (phosphorus limitation). The second set was a replica for each condition: B101-X3 (nitrogen limitation) and B101-X4 (phosphorus limitation).

The pH was set at 7.00 ± 0.05 and controlled by automatic addition of NaOH (1 M) or H_2_SO_4_ (1 M). Dissolved oxygen was maintained above 40% of saturation by varying the agitation speed. Two different mineral media were used in fed-batch experiments, as described above.

Culture samples were harvested periodically to measure cell dry weight (CDW); xylose, nitrogen, and phosphorus concentration; P(3HB) content; and residual biomass [Xr = CDW – P(3HB) in g/L].

### Gene Annotation

The genome sequence of *B. sacchari* (Alexandrino et al., 2015), privately available at RAST server (Aziz et al., 2008), was analyzed regarding the presence of genes related to phosphorus consumption, namely, the *pst* operon, and polyphosphate kinase genes. BLAST tool (Altschul et al., 1990) was used to confirm annotation and to compare protein sequences with *Escherichia coli*. Promoter sequences were predicted using Softberry BPROM (Solovyev and Salamov, 2011).

### Analytical Methods

#### Biomass Concentration

The cells from 10 ml of culture were harvested by centrifugation at 10,600 *g* and lyophilized in microtubes. Dry biomass was weighed using an Adventurer® Analytical balance (OHAUS, Parsippany, New Jersey, USA) and expressed as CDW (cell dry weight in grams per liter).

#### Xylose Determination

Xylose concentration was determined by HPLC. Samples were injected into an Ultimate 3000 HPLC (Thermo Fisher Scientific Inc., Waltham, MA, USA) equipped with an Aminex-HPX-87H (Bio-Rad Laboratories Inc., Hercules, California, USA). Separation occurred at 40°C with H_2_SO_4_ solution (5 mM) at a flow rate of 0.6 ml/min. The standard curve was constructed using D(+)xylose solutions (Merck KGaA, Darmstadt, Germany). A refractive index detector, Shodex RI-101 (Shodex, Munich, Germany), was applied for peak detection.

#### Ammonium Concentration

The ammonium concentration was determined with an ion-selective electrode. After alkalinization of the sample, a High Performance Ammonia Ion Selective Electrode Orion 9512HPBNWP (Thermo Fisher Scientific Inc., Waltham, MA, USA) coupled to an Orion™ 4-Star Plus pH/ISE Benchtop Multiparameter Meter (Thermo Fisher Scientific Inc., Waltham, MA, USA) was used to measure the ammonia gas formed. Ammonium amount was calculated from a (NH_4_)_2_SO_4_ standard curve containing up to 500 ppm of N.

#### Phosphorus determination

Phosphorus concentration was determined using the ascorbic acid colorimetric method (adapted from Rice et al., 2012). Briefly, a volume of reactive solution [20% (v/v) H_2_SO_4_ 6N, 20% (v/v) of ammonium molybdate 2.5% (w/v), and 20% (v/v) ascorbic acid 10% (w/v)] was added to an equal volume of supernatant samples. After incubation at 37°C for 1 h, absorbance was read at 820 nm.

#### Poly(3-hydroxybutyrate) Content Measurement

Ten milligrams of freeze-dried cells was propanolyzed (Riis and Mai, 1988). P(3HB) content was determined as described previously (Gomez et al., 1996) with an Agilent 7890A GC System (Agilent Technologies, Santa Clara, California, USA) equipped with an HP1 capillary column after sample split (1:10). Nitrogen (1.0 ml/min) was used as the carrier gas. Injector and flame ionization detector temperature were 250 and 300°C, respectively. The oven was programmed to maintain the temperature at 100°C for 1 min and then increase the temperature at a rate of 8°C /min up to 210°C, which was maintained for 15 min. Benzoic acid was used as the internal standard (Sigma-Aldrich, Saint Louis, Missouri, USA). P(3HB-*co*-3HV) (Sigma-Aldrich, Saint Louis, Missouri, USA) and medium-chain-length (C6–C12) PHA produced by *P. putida* ATCC 29347 from different fatty acids or by *Pseudomonas* sp. LFM046 from glucose were used as external standards.

## Results

### Growth Phase

**Figure 1:**
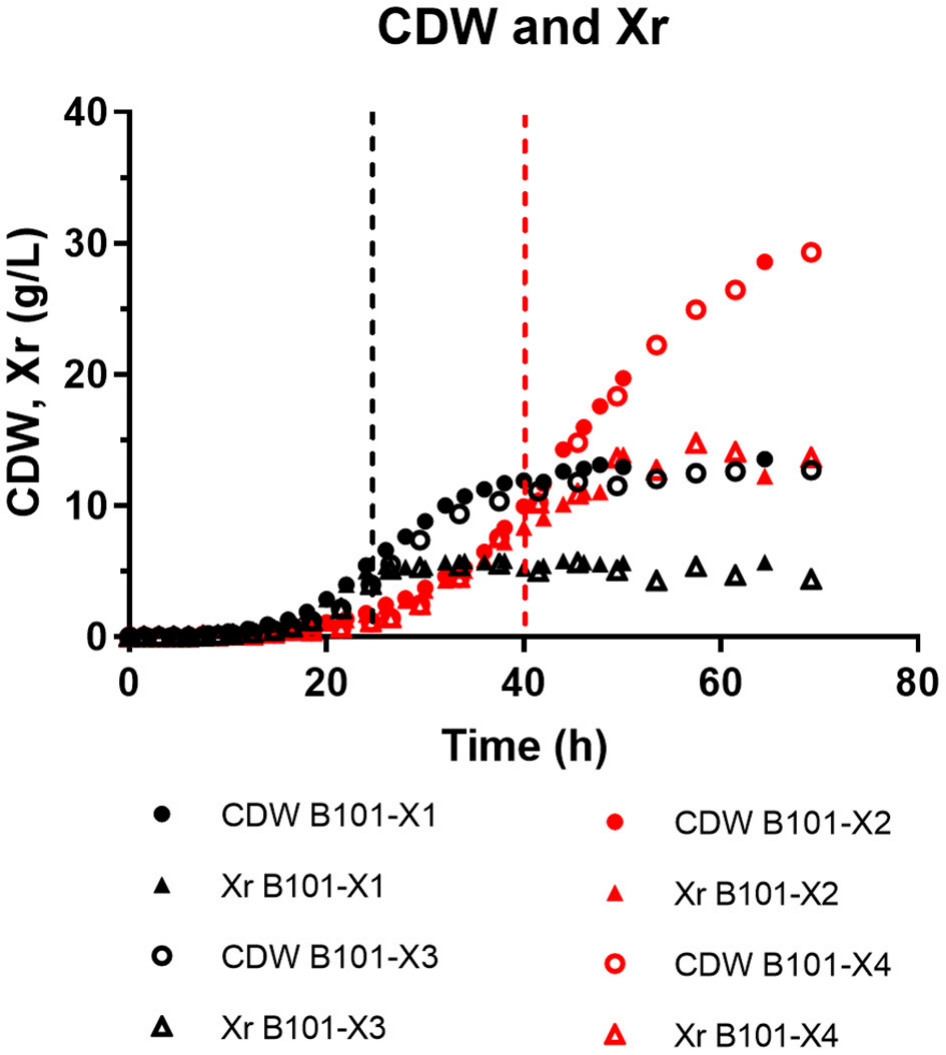
Cell dry weight (CDW) and residual biomass (Xr) (both in g/L) obtained for each fed-batch cultivation. B101-X1 and B101-X3 refer to experiments under nitrogen limitation (black dots/lines), while B101-X2 and B101-X4 refer to experiments under phosphorus limitation (red dots/lines).

**Figure 2:**
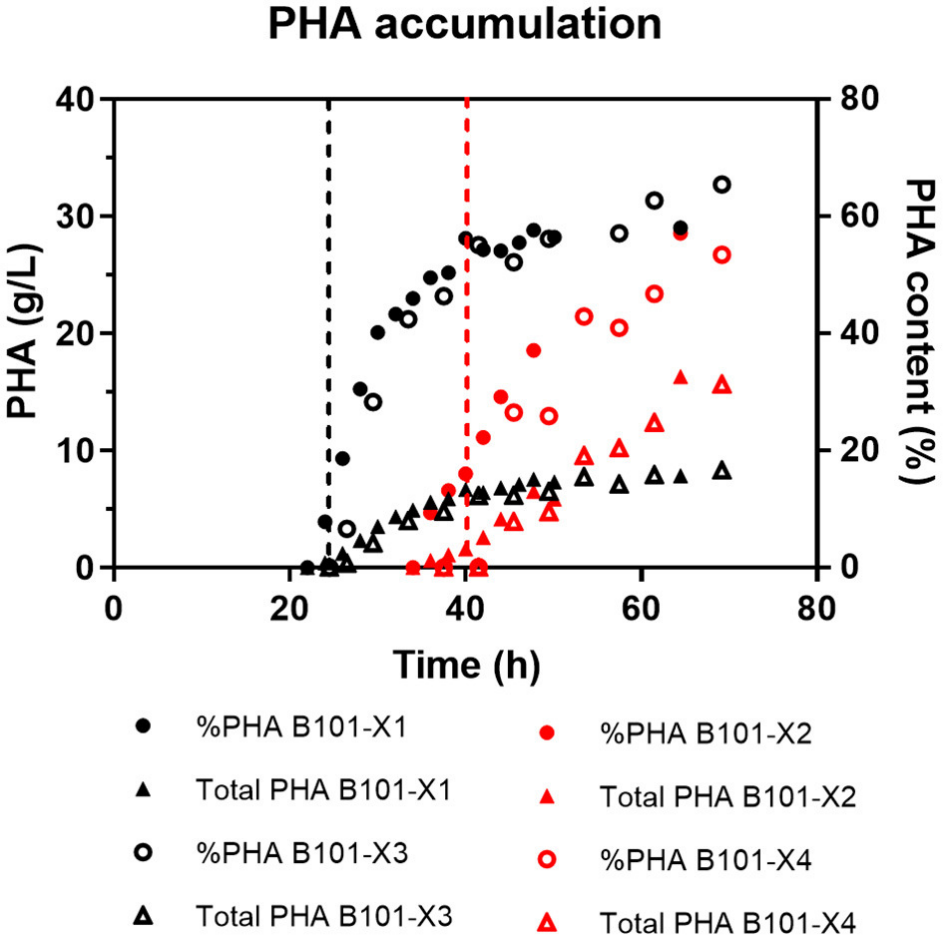
PHA (g/L, left y-axis) and content (%, right y-axis) obtained for each fed-batch cultivation. B101-X1 and B101-X3 refer to experiments under nitrogen limitation (black dots/lines), while B101-X2 and B101-X4 refer to experiments under phosphorus limitation (red dots/lines). Black dashed lines mark the start of the accumulation phase under nitrogen limitation, while red dashed lines mark the start of the accumulation phase under phosphorus limitation.

**Figure 3:**
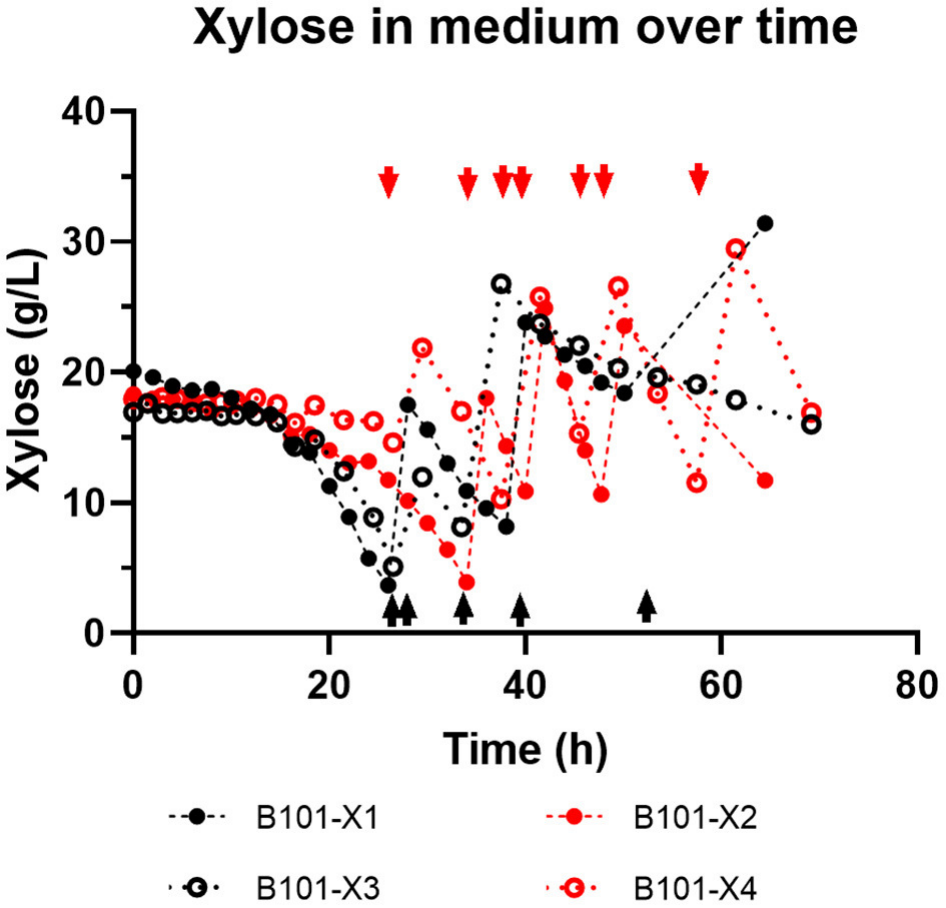
Xylose concentration (g/L) in medium over time obtained for each fed-batch cultivation. B101-X1 and B101-X3 refer to experiments under nitrogen limitation (black dots/lines), while B101-X2 and B101-X4 refer to experiments under phosphorus limitation (red dots/lines). Black dashed lines mark the start of the accumulation phase under nitrogen limitation, while red dashed lines mark the start of the accumulation phase under phosphorus limitation. Arrows indicate feeding instants.

**Figure 4:**
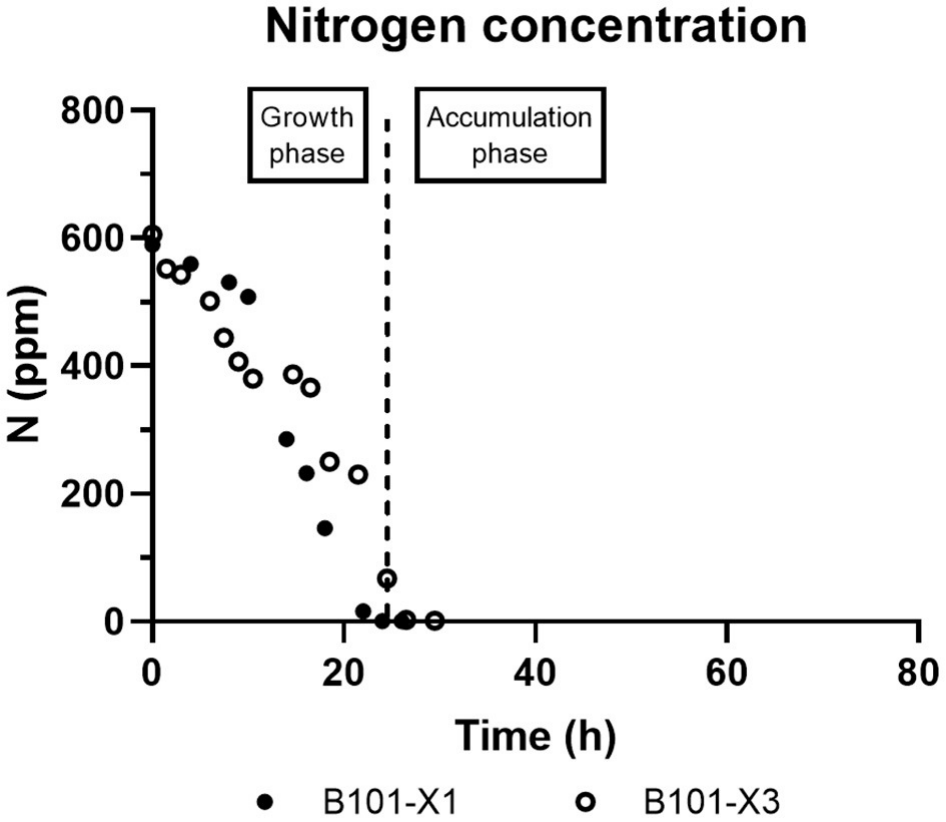
B101-X1 and B101-X3 nitrogen concentration over time.

In all experiments, P(3HB) accumulation was not detected during the growth phase (Figures 1, 2). B101-X1 and B101-X3 are replicas of nitrogen limitation condition, while B101-X2 and B101-X4 are replicas of phosphorus limitation condition. Xylose concentration in medium over time for each fed-batch is presented in Figure 3. Regarding nitrogen-limited fed-batches (B101-X1 and B101-X3), P(3HB) accumulation started after 24–26 h of cultivation when nitrogen concentration was almost depleted (2–12 mg/L) (Figures 2, 4). In these fed-batches, phosphorus quantification revealed that this nutrient was not limited during all the cultivation, although it was consumed after nitrogen depletion. Nitrogen to residual biomass yield (Y_Xr/N_) was calculated, representing around 7.5 (g/g).

**Figure 5:**
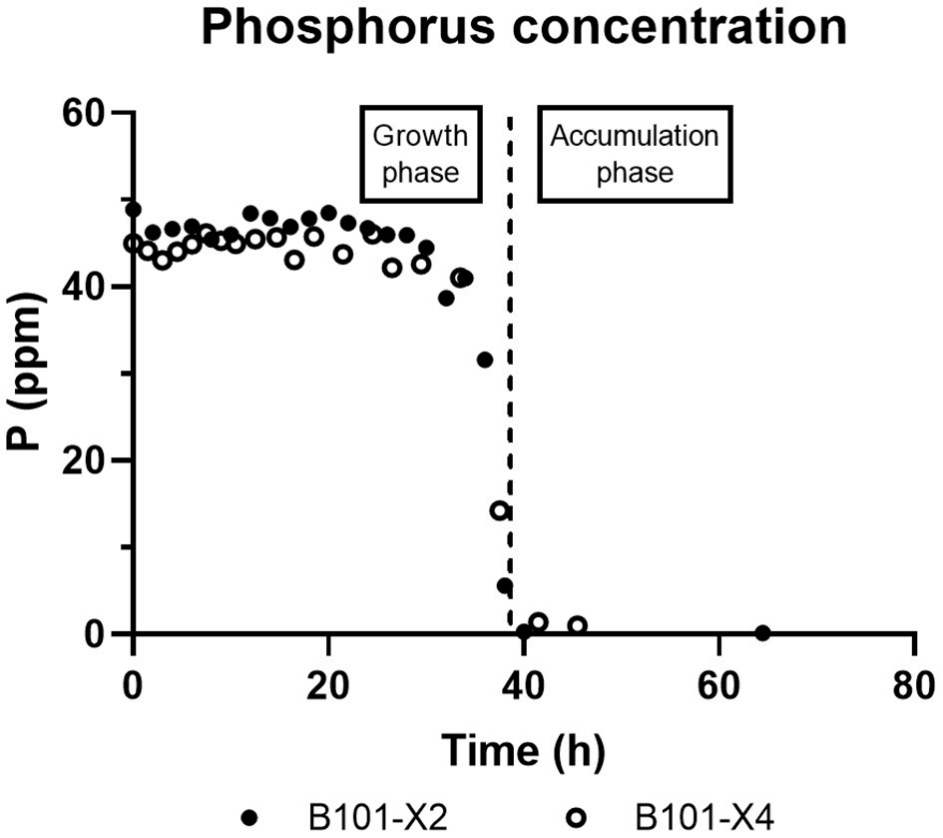
B101-X2 and B101-X4 phosphorus concentration over time.

**Figure 6:**
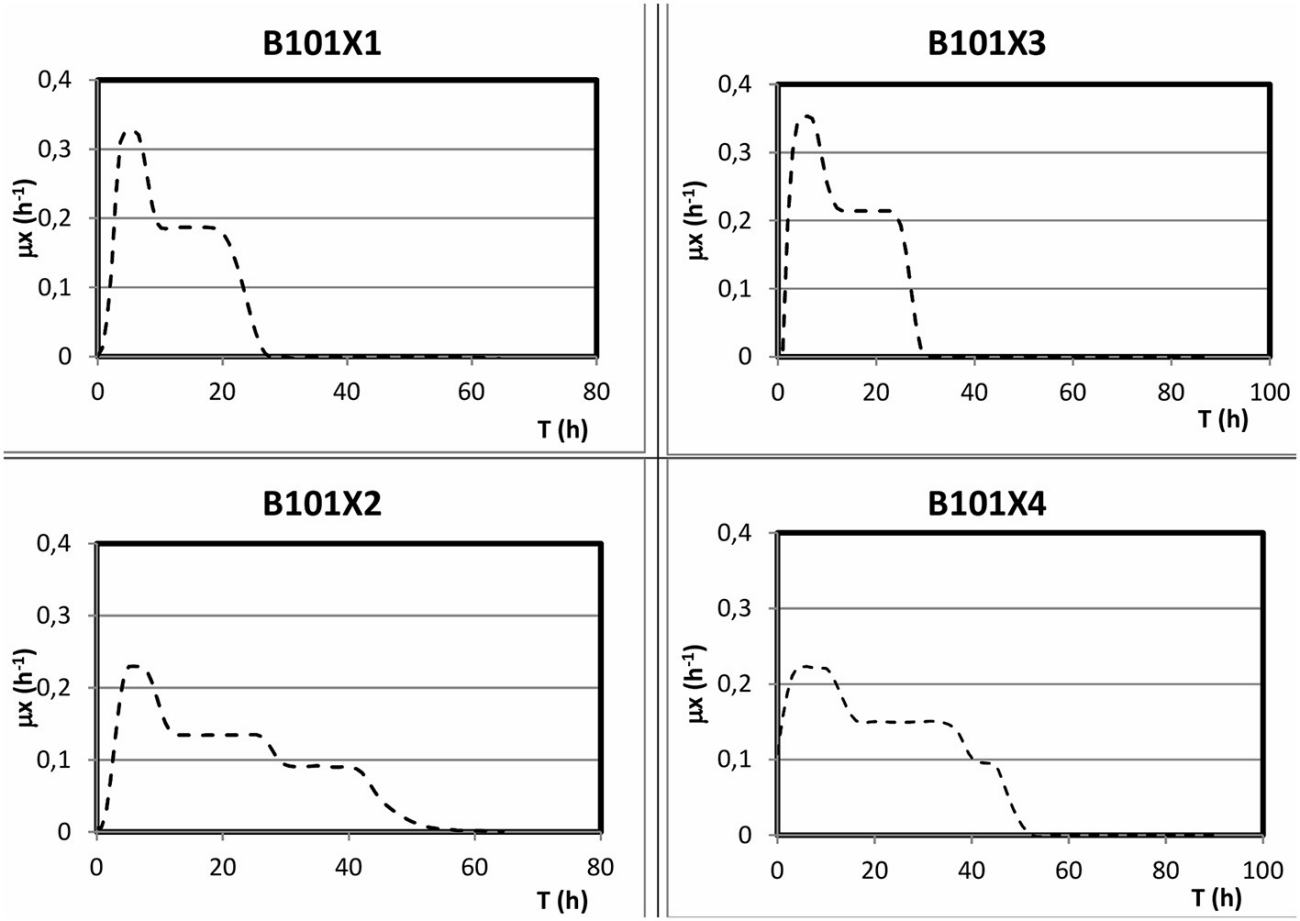
Representation of the obtained μ in different culture media.

Regarding phosphorus-limited experiments (B101-X2 and B101-X4), nitrogen quantification revealed that this nutrient was not limited during cultivation, as planned. Phosphorus concentration started to decrease after 20 h of batch, with phosphorus to residual biomass yield (Y_Xr/P_) around 80 g/g. P(3HB) accumulation started around 40 h of cultivation (Figure 2), when phosphorus concentration was *circa* 31 mg/L, indicating that this is the limiting concentration for *B. sacchari*. Phosphorus depletion was observed right after P(3HB) accumulation started (Figures 2, 5). Growth profiles were obtained, from which kinetic parameters were calculated. The variation profile of μ during the growth phase is represented in Figure 6.

### Poly(3-hydroxybutyrate) Accumulation Phase

Gas chromatography of the propyl-esters from samples was compared to chromatograms obtained from different standards ranging from 3HB to 3HDd (including unsaturated monomers). Only 3HB was detected (Supplementary Figure 1).

PHA accumulation phase started when cell growth stopped due to nitrogen (B101-X1 and B101-X3) (Figures 1, 2, 4) or phosphorus limitation (B101-X2 and B101-X4) (Figures 1, 2, 5). In this phase, acetyl-CoA generated from xylose metabolism was routed to PHA biosynthesis. Data obtained in two sets of experiments were used to calculate pseudo-stoichiometric and kinetic parameters of growth and PHA accumulation phases. These values are presented in Tables 2–4.

**Table 2:** Residual biomass yield from nitrogen or phosphorus (g/g) [Y_Xr/E_] from the tested conditions.

**Yield**	**Mean value ± Standard deviation**
Y_Xr/N_	7.63 ± 0.16
Y_Xr/P_	80

**Table 3:** Summary of data obtained in fed-batch experiments using *Burkholderia sacchari* LFM101, supplying xylose as the sole carbon source under different nutrient limitations to PHA accumulation.

**Limitation**	**Parameters**		
	**CDW (g/L)**	**P(3HB) (%)**	**Volumetric productivity (g/L·h)**
Nitrogen	13.10 ± 0.57	61.70 ± 5.23	0.12 ± 0.00
Phosphorus	29.25 ± 0.90	55.25 ± 2.62	0.24 ± 0.01

*CDW: cell dry weight (g/L); P(3HB): polymer content of CDW (% of CDW)*.

**Table 4:** P(3HB) yields from xylose (g/g) [Y_P(3HB)/xyl_] and specific P(3HB) production rates (1/h) [q_P_] from the tested conditions.

**Limitation**	**Phase 1**	**Phase 2**
**Y**_**P(3HB)/xyl**_ **(g/g)**		
Nitrogen	0.38 ± 0.00 (24–34 h)	0.28 ± 0.01 (34–48 h)
Phosphorus	0.36 ± 0.01 (40–69 h)	–
**q**_**P**_ **(1/h)**		
Nitrogen	0.09 ± 0.01 (24–34 h)	0.03 ± 0.01 (34–49.5 h)
Phosphorus	0.04 ± 0.00 (40–69 h)	–

*Nitrogen limited fed-batches presented two phases of biopolymer accumulation, while phosphorus-limited ones presented only one during all the experimental time. Each phase also presents a different q_P_. The time intervals of each phase are indicated in parentheses*.

Two-phases of P(3HB) production were observed in nitrogen-limited experiments (Figure 2), phase 1 from 23 to 34 h and phase 2 from 34 to 49 h. This was reflected by xylose to P(3HB) yields [Y_P(3HB)/xyl_] and specific rates of P(3HB) production (q_P_). In phosphorus-limited experiments, a single phase of P(3HB) production was observed during the entire experiment course (Figure 2), with a single Y_P(3HB)/xyl_ and q_P_.

### High-Affinity Inorganic Phosphate Transport and Polyphosphate Production Encoding Genes

*B. sacchari* genome was browsed using SEED viewer online tool (Overbeek et al., 2014). Table 5 lists the gene sequences found, as well as the encoded protein, its length, and identity to *E. coli* protein.

**Table 5:** Features of *B. sacchari* inorganic phosphate transport and polyphosphate production genes and protein identity to *E. coli* homologs.

**Gene**	**Encoded protein**	**Identity^a^ (%)**	**Protein length (aa)**
*pstS*	Phosphate-binding protein	62	343
*pstC*	Phosphate transport system permease protein	75	328
*pstA*	Phosphate transport system permease protein	74	298
*pstB*	Phosphate import ATP-binding protein	82	278
*phoU*	Phosphate-specific transport system accessory protein	48	234
*phoB*	Phosphate regulon transcriptional regulatory protein	58	233
*phoR*	Phosphate regulon sensor protein	37	437
*ppk1*	Polyphosphate kinase 1	36	706
*ppk2*	Polyphosphate kinase 2	62	375
*ppx*	Exopolyphosphatase	41	515

a *Identity with E. coli homologs*.

Regarding the operon arrangement, *pstS, pstC, pstA*, and *pstB* are arranged as an operon, as a simple promoter was predicted upstream *pstS*. Another promoter sequence was predicted upstream of *phoU* (inside *pstB* coding sequence), forming a *phoUB* operon. Finally, regulatory sequence *phoR* possesses its own promoter sequence. Interestingly, *ppk* and *ppx* genes were located downstream of *phoR* sequence. A close *in silico* examination revealed an rpoD17 binding site in *phoUB* and *ppx* putative promoter sequences and an rpoD16 binding site in *ppk* putative promoter sequence.

**Figure 7:**
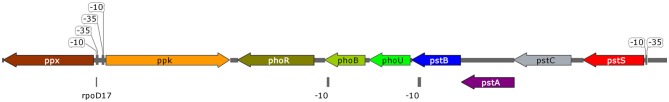
Scheme of *B. sacchari* LFM101 high-affinity phosphate consumption and polyphosphate production gene cluster.

Figure 7 represents the arrangement of the cluster composed by *pst* genes plus *ppk* and *ppx*. A gene encoding for polyphosphate kinase 2 (EC 2.7.4.1) was also annotated, although located at a different genomic loci.

## Discussion

### Growth Phase

A few bacterial strains are considered as efficient regarding growth on xylose, the most abundant sugar in hemicellulosic residues (Li et al., 2017). The study of different experimental conditions to convert xylose into fine chemicals is of great interest in order to define new perspectives to design and establish efficient bioprocess techniques. Phosphorus-limited experiments achieved higher CDW and biomass when compared to nitrogen-limited experiments.

From the obtained μ values, we can infer two (nitrogen limitation) or three (phosphorus limitation) different specific growth rates (Figure 6). In nitrogen-limitation condition (B101-X1 and X3), maximum specific growth rates were maintained for up to 6 h of cultivation around 0.34 1/h. After this period, μ decreased to values (around 0.20 1/h) that were maintained until the end of the growth phase. On the other hand, maximum specific growth rates in phosphorus limitation condition (B101-X2 and X4) reached 0.22 1/h, shifting to 0.14 1/h in the second growth phase and then finally to 0.09 1/h until the end of growth. It is important to notice that the second phase was longer under higher concentrations of phosphorus (B101-X1 and X3).

In a different study, when cultivated under lower phosphorus concentration, *B. sacchari* growth rate was 0.15 1/h (Guamán et al., 2018), lower than the herein obtained under higher phosphorus concentrations (B101-X1 and -X3), which suggests that phosphorus concentration was limiting growth.

In nitrogen-limited experiments, residual biomass yield from nitrogen was around 7.5 g/g, close to what was obtained for *B. sacchari* in a previous study by Rocha and co-authors (7.25 g/g) (Rocha et al., 2008). Also for the same strain, a similar yield value was cited by Gomez and co-authors (6.62 g/g) (Gomez et al., 1997). This Y_Xr/N_ value is similar to that obtained for *E. coli*, 8.00 (g/g) (Egli, 2015). Y_Xr/N_ values from 5 to 8 g/g were reported for *P. putida* IPT046 in bioreactor cultivations under different conditions of dissolved oxygen and nitrogen amounts (Diniz et al., 2004). Since *B. sacchari* is not able to fix nitrogen (Martínez-Aguilar et al., 2008), we can assume that this nutrient is used mainly for nucleic acids and protein biosynthesis.

Regarding phosphorus concentration in P-limited cultivations, KH_2_PO_4_ amount in the initial batch was calculated to achieve a residual CDW of 5 g/L, considering a Y_Xr/P_ value of 40 g/g (Taciro, 2008). As these conditions rendered an Xr value around 10 g/L (2-fold increase when compared to the expected value), we can assume that Y_Xr/P_ for *B. sacchari* is around 80 g/g, similar to what was previously obtained for *B. sacchari* supplied with sucrose, around 82 g/g (Gomez et al., 1997). The obtained Y_Xr/P_ is considerably higher when compared to *E. coli*, 33 g/g (Egli, 2015). Taciro (2008) mentions Y_Xr/P_ values from chemostats of 30–40 g/g under phosphorus limitation and 20–30 g/g under nitrogen limitation conditions for *P. putida* IPT046. Considering those Y_Xr/P_ values for *E. coli* and *Pseudomonas, B. sacchari* biomass composition presents lower phosphorus amounts. Also, as phosphorus is a significant component of nucleic acids, it should be noted that *B. sacchari* genome size is 7.2 Mb (Alexandrino et al., 2015), *E. coli* K-12 MG1655 genome size is 4.6 Mb (Blattner et al., 1997), and *Pseudomonas* LFM046 genome size is 6.0 Mb (Cardinali-Rezende et al., 2015). In addition, phospholipids are also important components synthesized from phosphorus.

Interestingly, phosphorus-limited experiments presented residual biomass production (Xr) during the accumulation phase (Figure 1), even after phosphorus depletion in the culture media, albeit severely reduced when compared to Xr increase during the growth phase. We suggest that the polyphosphate produced during the growth phase was then degraded and sustained Xr increase after phosphorus depletion in the culture media. Also, phosphorus consumption after nitrogen depletion in nitrogen-limited experiments strongly suggests that *B. sacchari* accumulates polyphosphate, similar to what was found previously by Gomez et al. (1997). Nevertheless, it is also important to mention that the seed culture was grown in a phosphorus-rich medium (MM1), probably using *pit*-mediated transport, a phosphate low-affinity transport system. Cells that were grown with P-limitation may already have a higher adaptation requirement at first, i.e., activation of the *pst*-mediated transport system.

### *B. sacchari* Phosphate Transport and Polyphosphate Metabolism Gene Cluster

Genome analysis revealed that a complete *pst* locus is present in *B. sacchari* genome, together with *ppk1* and *ppx* located downstream of *pst* locus. *ppk2* gene was located elsewhere in the chromosome. A similar gene arrangement was found in *Burkholderia xenovorans* LB400, *Ralstonia solanacearum* GMI1000, *Burkholderia ambifaria* AMMD, and *R. eutropha* JMP134 (genome sequences available for comparison at RAST server). A similar operon organization (number and location of gene promoters, i.e., regulation) was described in *Pseudomonas aeruginosa* (Munévar et al., 2017). These genes are part of the PHO regulon, regulated by the concentration of phosphate in the medium (Santos-Beneit, 2015). Phosphate uptake in *E. coli* occurs following two kinetically different systems: high-affinity (Pst) and low-affinity (Pit) systems (Medveczky and Rosenberg, 1971; Willsky and Malamy, 1976). The Pst system is active in phosphorus-limiting conditions and, as such, is of greatest interest to our study. The Pst system is a conventional ABC transporter, in which PstS binds to extracellular phosphate, PstA and PstC are permeases that channel phosphate through the periplasmic space (Webb et al., 1992), and PstB binds to ATP, which is the energy source for the transport (Chan and Torriani, 1996). This transport system was previously reported in a *Burkholderia* strain (Ruiz-Lozano and Bonfante, 1999). Regarding *phoB* and *phoR* gene products, PhoB and PhoR are part of a two-component system that regulates PHO gene expression. PhoR sensor kinase autophosphorylates and phosphorylates PhoB in low-phosphate concentration. PhoB-P then promotes the expression of PHO genes (Santos-Beneit, 2015). Under phosphate excess, regulator protein PhoU is known to repress PHO genes expression via dephosphorylation of PhoB-P.

Putative promoter sequences were identified for *ppk1, ppk2*, and *ppx*, the central genes in polyphosphate formation (*ppk1* and *ppx*) and degradation (*ppk2* and *ppx*). As found in *P. aeruginosa, ppk1* and *ppx* are adjacent and in opposite directions (Zago et al., 1999), suggesting a differential expression regulation. Ppk1 protein is involved in the formation of polymer from ATP, while Ppk2 encoding gene, which was annotated in a different genomic *loci*, is typically found in *P. aeruginosa* and associated with the synthesis/degradation of polyphosphate from/to GTP or ATP (Zhang et al., 2002). Ppx degrades polyphosphate into orthophosphate, and its encoding sequence was first described in *E. coli* forming an operon with *ppk1* protein (Akiyama et al., 1993). We identified a putative promoter sequence in *B. sacchari*'s *ppx*, which indicates differential regulation.

Together, the increased Y_Xr/P_ (higher to what has been presented in the literature) and genomic evidence suggests that *B. sacchari* accumulates polyphosphate granules, as firstly suggested by Gomez and co-workers (Gomez et al., 1997).

### Poly(3-hydroxybutyrate) Accumulation Phase

P(3HB) contents were similar in both tested conditions (55–61% of cell dry weight), comparable to what was reported in previous studies using *R. eutropha* (Kim et al., 1994; Ryu et al., 1997).

Nitrogen limitation yielded approximately equal P(3HB) content (7.8 g/L). In contrast, phosphorus limitation yielded higher P(3HB) amount (*ca*. 16 g/L). This reflected in the volumetric productivity of P(3HB) in this strategy [0.25 gP(3HB)/L·h], which can be attributed to higher cell growth sustained by greater levels of nitrogen in MM3.

Under nitrogen limitation, two different biopolymer accumulation phases were observed. The first one presented a Y_P(3HB)/xyl_ (0.38 g/g) representing 79% of the maximum theoretical value (0.48 g/g), calculated considering that 6 mol of xylose is converted to 5 mol of 3HB. The second accumulation phase yield (0.275 g/g) represents 57% of the maximum theoretical value.

In comparison, phosphorus-limited experiments presented a single accumulation phase during the experiment time. The obtained Y_P(3HB)/xyl_ value, 0.36 g/g, corresponds to 74% of the maximum theoretical value. Although only one phase was observed, these cultivations required longer time to start P(3HB) accumulation, suggesting that longer experiments could clarify the accumulation profile of *B. sacchari* in this condition.

Nitrogen is used by bacterial cells to synthesize nucleic acids and proteins, while phosphorus is converted to nucleic acids and phospholipids. We can note that in the accumulation phase, q_P_ values are 1-fold reduced in phosphorus-limited experiments when compared to nitrogen limitation condition.

Although under phosphorus limitation conditions *B. sacchari* achieved higher P(3HB) production in g/L, P(3HB) production required more time, almost 20 h, to achieve an equal P(3HB) content [%P(3HB)] when compared to the nitrogen limitation condition. A similar situation was described in a study investigating the effect of phosphorus limitation on PHA production by activated sludge biomass (Chinwetkitvanich et al., 2004). The phosphorus consumption of *B. sacchari* showed that this nutrient was substantially consumed after 20 h of cultivation, reaching depletion shortly after this period. The same profile was reported by Ryu and co-workers when studying PHA accumulation by *R. eutropha* (Ryu et al., 1997).

In this study, P(3HB), the most-studied PHA, was produced from xylose. P(3HB) characteristics include high crystallinity, stiffness, and brittleness. *B*. *sacchari* is also capable of incorporating other monomers as 3HV (Silva et al., 2002), 3HHx (Mendonça et al., 2017), and 4HB (Cesário et al., 2014; Miranda De Sousa Dias et al., 2017) when adequate co-substrates are supplied; therefore, studies combining xylose and co-substrates feeding may benefit from the results here presented, contributing to reduce costs of production by using xylose from hemicellulosic residues.

## Conclusions

This work describes, for the first time to our knowledge, the effects of two different nutritional limitations on the growth and P(3HB) accumulation by the non-model *bacterium B. sacchari* from xylose solely. Higher μ_max_ were observed under nitrogen-limiting conditions, indicating that an increased phosphorus concentration is a defining factor to achieve higher growth rates on xylose. Although phosphorus limitation (nitrogen excess) resulted in higher CDW, and thus higher P(3HB) concentration, in g/L, the biopolymer accumulation phase started 20 h after nitrogen limitation conditions. To avoid this delay, the seed cultures could be previously adapted to diminished phosphorus concentrations. Further transcriptomics studies, including PHO regulon expression assessment, would contribute to elucidate the metabolic pathways responsible for the observed physiological phenomenon. Since *B. sacchari* is considered as a promising microbial cell factory for the biotechnological production of PHA and xylitol (Raposo et al., 2017), this work may contribute to the establishment of cheaper and sustainable production of polyhydroxybutyrate from agroindustrial by-products. Considering the context of integrating PHA production to a sugar and ethanol biorefinery, the use of xylose, not really fermented by ethanologenic yeasts, can represent an important step toward sustainability of the process. Results presented here contribute to the knowledge on the conditions appropriate do promote the best efficiency in converting xylose to P(3HB) in this model.

## Data Availability Statement

The datasets generated for this study are available on request to the corresponding author.

## Author Contributions

LS, JG, and MT jointly conceived and supervised the study. EO-F and JS performed the experiments. EO-F and MM prepared the initial version of this manuscript. All authors analyzed the data, read, improved, and approved the final manuscript.

### Conflict of Interest

The authors declare that the research was conducted in the absence of any commercial or financial relationships that could be construed as a potential conflict of interest.
