# Supplementary material for: Investigating Nutrient Limitation Role on Improvement of Growth and Poly(3-Hydroxybutyrate) Accumulation by Burkholderia sacchari LMG 19450 From Xylose as the Sole Carbon Source
Source: Front Bioeng Biotechnol. 2020 Jan 8;7:416. doi: 10.3389/fbioe.2019.00416 (PMC6960187; doi:10.3389/fbioe.2019.00416)
Supplement: Supplementary file 1 [file Data_Sheet_1.PDF]

Supplementary Material

1.1 Supplementary Figure

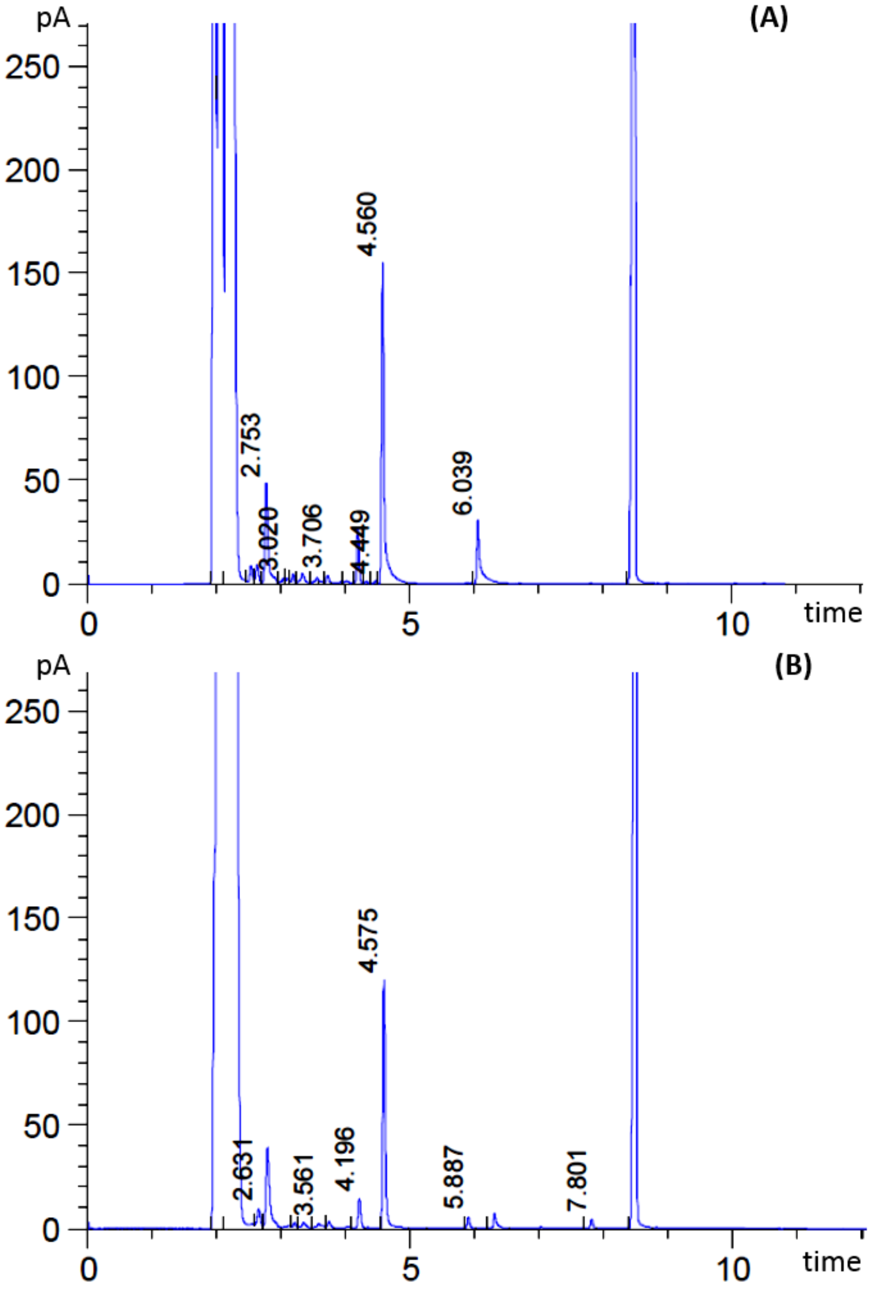

Supplementary Figure 1. Gas chromatography of propyl-esters of standards (A) and samples obtained in bioreactor assays with *Burkholderia sacchari* LMG19450 (B) - FID signal (pA) versus

time (minutes). From (A), we can infer that 3HB was detected at 4.560 min and 3HV at 6.039 min. Obtained samples present only 3HB peak, as shown in (B).
